# Study on the differentiated impact of climate change on plague epidemics in Northern and Southern China, 1912–1949

**DOI:** 10.1371/journal.pntd.0014036

**Published:** 2026-03-06

**Authors:** Lei Zhang, Shuyan Yin, Miao Ge, Lin Pang

**Affiliations:** 1 School of Geography and Tourism, Shaanxi Normal University, Xi’an, Shaanxi, China; 2 Chinese academy of sciences institute of earth environment, Key Laboratory of Loess Science, Key Laboratory of Aerosol Chemistry and Physics, Xi’an, Shaanxi, China; University of Virginia School of Medicine, UNITED STATES OF AMERICA

## Abstract

Based on plague disaster and climate data from China between 1912 and 1949, this study comprehensively employed the Mann-Whitney U test, mutation test, and optimal parameter geographic detector to investigate the relationship between plague epidemic characteristics and climate change across different geographic regions. Findings reveal significant spatiotemporal divergence in plague epidemics between northern and southern China: Southern plague exhibits a clearly defined “high-amplitude stable decline” trend, while northern plague shows a slow downward trajectory amid intense fluctuations, lacking a significant linear trend. Moreover, all three plague hotspots highly overlap with natural reservoirs. This divergence stems from fundamentally different climate-driven mechanisms in the north and south, with interactive detection indicating that synergistic effects between dual factors generally outweigh single-factor impacts. Northern plague is jointly controlled by precipitation fluctuations and thermal variations, primarily driven by the interaction between annual precipitation and trends in annual mean high temperatures (q-value: 31.46%); In contrast, southern plague is more sensitive to warming transitions in the climate system, primarily governed by the synergistic effects of annual temperature difference variations and trends in low temperatures, precipitation, and mean temperature (q-values: 38.44%, 34.92%, and 34.77%). Spatio-temporal coupling analysis further reveals that climate abruptions act as temporal triggers for epidemic shifts: Northern plague exhibits delayed peaks 1–2 years after precipitation abruptions, while Southern plague frequency declines during high-temperature abruptions. Spatially, high-value zones of Northern annual high-temperature trends form ecological barriers segmenting adjacent hotspots, whereas Southern low-value zones of annual temperature difference trends correspond to plague hotspots. By elucidating historical variations in plague sensitivity to climate fluctuations, this study provides crucial historical evidence and reference for contemporary plague surveillance and public health risk assessment under climate change.

## Author summary

The period from 1912 to 1949 coincided with the third major plague pandemic. Compared to the stringent control measures implemented by the Chinese government after the nation’s founding in 1949, the plague epidemics during this era more accurately reflected the natural characteristics of the disease. This provides a unique window for studying the inherent relationship between epidemics and climate. Through coupled analysis of plague epidemics and climate variations from 1912 to 1949, this study reveals distinct spatiotemporal patterns in plague distribution between northern and southern China. These differences stem from varying climatic drivers and ecological contexts. Abrupt climate shifts act as ‘triggers’ that periodically alter the spatial distribution of plague epidemics, while long-term climate trends shape the geographical boundaries of plague prevalence. This research deepens our understanding of the natural plague-climate relationship, providing scientific basis for assessing plague risks under current and future climate conditions and formulating control strategies.

## 1. Introduction

The relationship between climate change and health is a significant global concern. Global warming may facilitate the frequent emergence of novel infectious diseases such as COVID-19 and monkeypox, or heighten the risk of resurgence for previously eradicated or suppressed diseases, including plague [1]. Whether plague will re-emerge as a pandemic amid climate change has become a focal point for scholars. Consequently, a thorough understanding of historical plague patterns, particularly their key drivers, holds significant practical importance for plague surveillance and prevention.

Climate change can directly impact the habitats of reservoir animals (such as marmots and rodents), influence the growth and development of vectors (fleas), and affect the maturation and reproduction of plague pathogens, thereby indirectly affecting the occurrence and spread of plague [2–4]. Numerous studies have demonstrated that climatic factors such as temperature and precipitation, along with their variability, significantly influence plague. Unravelling these complex relationships is crucial for understanding disease dynamics and forecasting future risks, though such relationships exhibit high complexity and regional heterogeneity [5–7]. Research indicates that in northern China, pneumonic plague predominates, primarily transmitted via respiratory droplets, characterised by rapid onset and extensive spread; whereas southern regions predominantly experience bubonic plague, primarily transmitted via flea bites, characterised by rapid spread and more severe consequences [8,9]. Xu’s research indicates that in northern China, plague intensity increases with rising precipitation levels; conversely, in southern regions, plague intensity typically decreases with increased precipitation [10,11]. By comparing plague epidemiology, Xu identified temperature and precipitation variations as the primary factors causing differing peak seasons between northern and southern China, with northern regions exhibiting greater sensitivity to precipitation [12]. This regional specificity indicates nationwide investigations into plague determinants must be conducted regionally. Zhao’s research revealed that excessive and insufficient precipitation increase plague risk, with northern regions facing significantly higher risks than southern areas [13]. The regional specificity of these scholarly findings underscores the necessity for regionally differentiated approaches when investigating plague determinants.

During the Republic of China period (1912–1949), military factional warfare, economic depression, and social upheaval precipitated frequent epidemics [14]. Cholera ranked alongside plague and smallpox as one of the period’s three most virulent infectious diseases [15]. The period from 1912 to 1949 fell within the third major plague pandemic. Compared to the stringent control measures implemented by the Chinese government after the nation’s founding in 1949, the plague epidemics during this era more accurately reflected the natural characteristics of the disease. This period thus offers a unique window for studying the inherent relationship between epidemics and climate. It quantitatively analyses the contribution levels and underlying mechanisms of various climatic factors in plague epidemics, deepening our understanding of the natural relationship between plague and climate. Does the driving mechanism of climate factors on plague exhibit significant regional variation? The findings provide a scientific basis for assessing plague risks under current and future climatic conditions and formulating prevention and control strategies.

## 2. Materials and research methods

### 2.1. Data sources

Plague data is sourced from the Compilation of Historical Materials on Epidemic Disasters in China over Three Millennia (Republic of China Volume) [16]. To enhance data reliability and universality, plague data underwent three-pronged calibration prior to subsequent analysis: 1. Multi-source calibration of plague data. By integrating multiple historical sources—including official histories, local gazetteers, archives, imperial records, literary collections, medical case studies, and modern periodicals—we cross-verified duplicate entries for the same epidemic across different sources to ensure each outbreak was counted only once in the statistics. Entries explicitly documented as “plague” were rigorously selected, while records of other diseases with ambiguous definitions or unclear classifications were excluded. 2. Spatio-temporal normalization. The study period was strictly defined as January 1, 1912, to September 30, 1949. County-level administrative units served as the basic spatial unit. Historical place names were mapped to the administrative boundaries of the Republic of China era and spatially linked with modern Geographic Information Systems (GIS) to ensure consistency of geographic units across the 38-year time series. 3. Data Quality Assessment and Descriptive Statistics. The mean annual plague incidence rate for county-level units nationwide was 73.184, with a standard deviation of 33.047. The maximum value was 180, the minimum was 29, and the range was 151. The mean annual plague incidence rate for county-level units in northern regions was 23.868, with a standard deviation of 18.344, a maximum value of 79, a minimum value of 4, and a range of 75. The mean annual plague incidence rate for county-level units in southern regions was 49.316, with a standard deviation of 24.252, a maximum value of 101, a minimum value of 6, and a range of 95. Plague data are available in S1 Data. The division between northern and southern China primarily follows the natural geographical boundary of the Qinling-Huai River line, while also considering the integrity of cultural and economic regions [17]. This division not only marks the climatic boundary between subtropical and warm temperate zones but also serves as an ecogeographic barrier for the distribution of plague’s primary hosts [18]. In summary, through rigorous multi-source calibration and spatiotemporal normalization, this study establishes a long-term plague data foundation with high reliability and consistency, providing robust data support for subsequent in-depth exploration of climate-disease dynamics.

The base map data for this study is sourced from the standard map, available at https://www.webmap.cn/mapDataAction.do?method=forw&datasfdafd=%253Fmethod%253Dforw%2526resType%253D5%2526dName%253D%2525E6%2525AF%252594%2525E4%2525BE%25258B%2525E5%2525B0%2525BA%2526format%253Dcombox%2526name%253DidMapScale%2526value%253D1000000%2526secClass%253D%2525E5%252585%2525AC%2525E5%2525BC%252580. The base map has not been modified in any way. Climatic data were downloaded from the National Centre for Earth System Science Data (https://www.geodata.cn/main/), utilising monthly temperature and precipitation grid datasets dating from 1901 onwards. This dataset was generated for China using the Delta spatial downscaling method, based on CRU global 0.5° data and WorldClim high-resolution data. 496 independent meteorological stations have validated it and effectively reflect the spatial distribution of meteorological elements in China [19,20]. Data from 1912 to 1949 were extracted and subjected to visualisation and trend analysis within ArcMap Pro 3.3. The selected climatic factors comprised: (X1) annual mean precipitation, (X2) annual mean temperature, (X3) annual mean high temperature, (X4) annual mean low temperature, (X5) annual mean temperature difference, (X6) trend in annual precipitation, (X7) trend in annual mean temperature, (X8) trend in annual high temperature, (X9) trend in annual low temperature, and (X10) trend in annual temperature difference.

### 2.2. Research methods

(1) Mann-Whitney U test

The Mann-Whitney U Test, also known as the Mann-Whitney Rank Sum Test, is a statistical method proposed jointly by H.B. Mann and D.R. Whitney in 1947 for testing between two independent samples [21]. Its fundamental principle involves first hypothesising that the two sample groups originate from two populations identical in all respects except their means. Subsequently, based on the test results, it determines whether a significant difference exists between the means of these two populations, thereby establishing the statistical significance of this hypothesis.

(2) Mutational analysis

Mutational analysis is frequently employed in examining long-term climate change sequences, with its specific outcomes indicated by the trends of UF and UB curves [22]. The UF curve reflects the overall trend, while the intersection of UF and UB curves indicates a break. At a significance level of 0.05, U₀.₀₅ = ±1.96. If the intersection lies between the critical lines (±1.96) and the trend of the UF curve beyond this point exceeds the critical line, this intersection is deemed the starting point of the break.

(3) Mann-Kendall (M-K) trend test and sen slope

The non-parametric M-K test analyses long-term monotonic trends (increasing, decreasing, or no trend) in climate data. This method is independent of data distribution, robust to outliers, and suitable for long-term time series analysis [23]. The Sen slope further quantifies the intensity and direction of climate element trends, with the median slope indicating trend magnitude (positive values signifying increase, negative values signifying decrease). In contrast, its absolute value reflects the scale of change. Corresponding calculations and visualisations for multidimensional raster data were performed using ArcGIS.

(4) Optimal Parameter Geodetector (OPGD)

The OPGD model enhances the accuracy and interpretability of the original Geodetector model by selecting optimal discretisation algorithms and parameters [24]. Five discretisation methods were employed in this study: Selective Natural Breakpoint Classification, Quantile Classification, Standard Deviation Classification, Equal Interval Classification, and Geometric Interval Classification; the specific classification settings ranged from 3 to 8. The OPGD is particularly suitable for this study due to its high compatibility with historical data characteristics. Since it does not require the original data to satisfy the assumption of normal distribution, it effectively mitigates interference caused by historical data heterogeneity. Consequently, it can efficiently capture the complex nonlinear interactions between climatic factors and plague.

## 3. Results and analysis

### 3.1. Spatiotemporal characteristics of plague epidemics

#### 3.1.1. Decadal characteristics.

**Fig 1:**
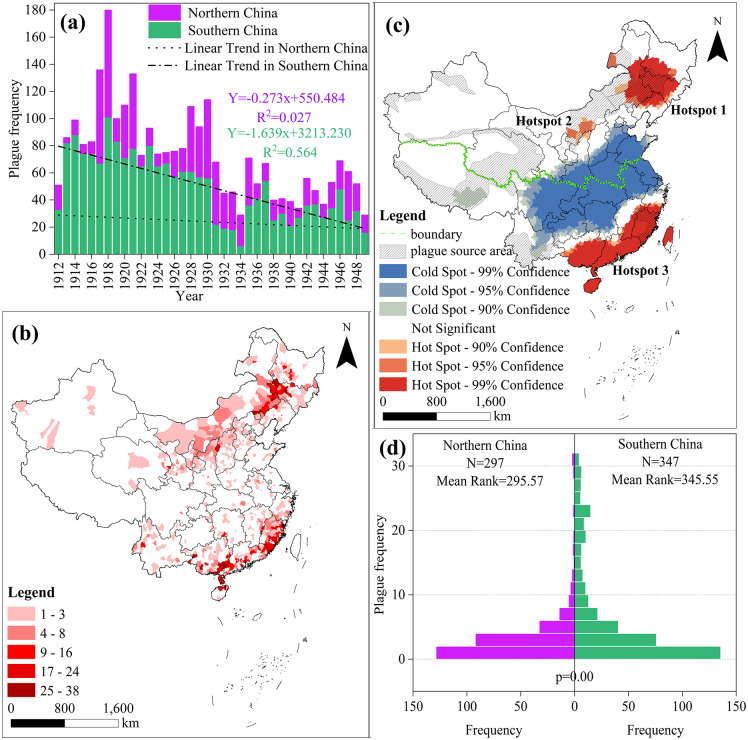
Spatiotemporal distribution and cluster analysis of plague in China. The data used in this study is from the National Earth System Science Data Center (http://www.geodata.cn). The base map data for this study is sourced from the standard map, available at https://www.webmap.cn/mapDataAction.do?method=forw&datasfdafd=%253Fmethod%253Dforw%2526resType%253D5%2526dName%253D%2525E6%2525AF%252594%2525E4%2525BE%25258B%2525E5%2525B0%2525BA%2526format%253Dcombox%2526name%253DidMapScale%2526value%253D1000000%2526secClass%253D%2525E5%252585%2525AC%2525E5%2525BC%252580. The base map has not been modified in any way.

The cumulative frequency of plague outbreaks in China during the 38 years of the 1912–1949 is depicted in Fig 1a, where purple indicates cumulative plague frequency in northern China and green denotes cumulative frequency in southern China. The nationwide peak occurred in 1918 with 180 outbreaks. Northern China experienced epidemic peaks in 1917–1921, 1928–1931, and 1946–1948, corresponding to four major plague pandemics in northern China’s history: the 1917–1918 outbreak in western Inner Mongolia and Shanxi; the 1920–1921 outbreak in Northeast China; the 1930–1931 outbreak in northern Shaanxi and Shanxi; and the 1946–1948 outbreak in Jilin and Inner Mongolia. In southern China, plague incidence can be divided into two distinct phases, separated by 1934: an earlier high-incidence period with an average frequency of 62.682 cases, and a later low-incidence period averaging only 30.937 cases. Although plague incidence in the north exhibits a declining trend, the rate of decrease is extremely slight and gradual. The slope is -0.273, indicating an average annual reduction of approximately 0.27 units. With R² = 0.027, merely 2.7% of the variation can be explained by the ‘linear decline’ pattern. Local control measures and climate change fluctuations may more significantly influence actual data. Southern plague incidence is characterised by ‘large magnitude and stable trend’, exhibiting a clear decline pattern (y = -1.639x + 3213.230). The regression coefficient (slope) of -1.639 indicates an annual average decrease of approximately 1.64 units in plague frequency over the time series. R² = 0.564 (P < 0.01) exceeds the highly significant threshold, confirming a pronounced and relatively stable decline with distinct temporal drivers.

#### 3.1.2. Spatial characteristics.

The provincial spatial distribution of plague exhibits both extensive geographical coverage and regional concentration (Fig 1b). In northern regions, provincial distribution centres on Inner Mongolia, Jilin, Shanxi, and Heilongjiang, with these four provinces accounting for 68.31% of county-level plague incidence (Inner Mongolia 28.82% > Jilin 18.48% > Shanxi 13.42% > Heilongjiang 7.59%). In southern regions, provincial distribution centres on Fujian, Guangdong, Guangxi, and Yunnan, with these four provinces accounting for 88.07% of county-level plague occurrences (Fujian 39.71% > Guangdong 23.54% > Guangxi 19.45% > Yunnan 5.37%).

The spatial distribution of plague at the county level across northern and southern China exhibits distinct hotspot and coldspot patterns (Fig 1c). Hotspot 1 and hotspot 2 are located in the northern region, while hotspot 3 is situated in the southern region. Hotspot 1 is centred at the junction of Heilongjiang, Jilin, and Inner Mongolia, with Tongliao City, Da’an City, and Tongyu County as its core areas. Hotspot 2 is located in the agro-pastoral transition zone spanning western Inner Mongolia and northern Shaanxi, centred on Ordos. Hotspot 3 primarily encompasses the southern coastal regions of Guangxi Province, Guangdong Province, and the southeastern coastal areas of Fujian Province. Furthermore, overlaying plague foci with hotspot/coldspot distributions reveals substantial overlap between all hotspot regions and plague foci (Fig 1c).

#### 3.1.3. Grouping test for plague.

The spatio-temporal distribution and hotspot/coldspot patterns of plague indicate distinct characteristics between northern and southern China. To further validate these regional differences, the Mann-Whitney U Test was applied to compare northern and southern plague patterns, with results presented in Fig 1d. The null hypothesis posited no significant difference between northern and southern China. The progressive significance level p = 0.00 < 0.05 indicates the null hypothesis is rejected, with average ranks of 295.57 and 345.55, demonstrating substantial disparity. Combined with the significance level p and average ranks, this confirms a significant difference between northern and southern China’s plague patterns. Consequently, studies examining the impact of climate change on China’s plague incidence should distinguish between northern and southern regions. To identify the climatic transition points associated with the differentiation characteristics between northern and southern plague strains, further research is still required.

### 3.2. Characteristics of climate change in China from 1912 to 1949

#### 3.2.1. M-K Test.

This study employs the M-K trend test to analyze climate trends for 38a across different regions in northern and southern China, aiming to identify overall climate change patterns. Results are presented in the Table 1. Combining Z-statistics and significance p-values reveals that X2 annual mean temperature, X3 annual mean high temperature, and X4 annual mean low temperature exhibit highly significant (p < 0.01) upward trends in both northern and southern regions. In the northern region, X1 annual mean precipitation exhibits a non-significant (p > 0.05) upward trend, while X5 annual mean temperature difference shows a significant (p < 0.05) upward trend. Conversely, in the southern region, both X1 annual mean precipitation and X5 annual mean temperature difference display non-significant (p > 0.05) downward trends. To effectively pinpoint critical turning points in the aforementioned climatic factor trends, a change-of-trend test was subsequently applied for detailed investigation.

**Table 1:** M-K test results for climate variables in northern and southern China.

Region	Factor	Statistical Z value	P value	Variation trend	Region	Factor	Statistical Z value	P value	Variation trend
Northern China	X1	1.408	0.159	upward trend	Southern China	X1	-0.478	0.633	downward trend
	X2	4.576	0.000	upward trend		X2	4.476	0.000	upward trend
	X3	4.274	0.000	upward trend		X3	4.400	0.000	upward trend
	X4	4.174	0.000	upward trend		X4	2.942	0.003	upward trend
	X5	1.986	0.047	upward trend		X5	0.075	0.940	upward trend

#### 3.2.2. Mutational analysis.

**Fig 2:**
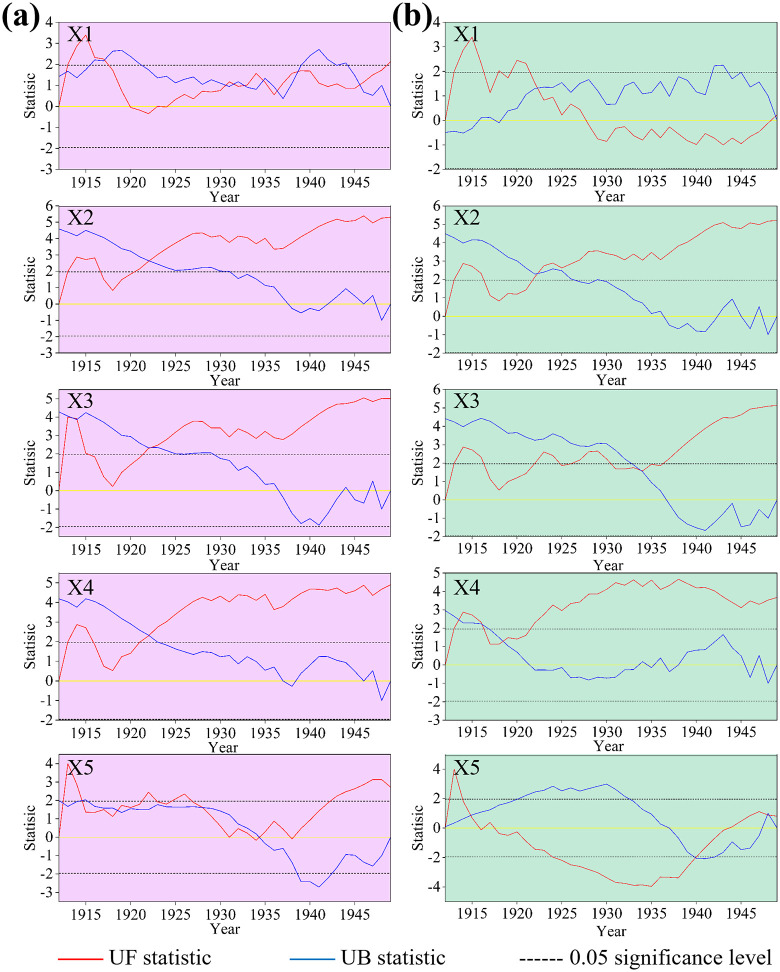
Test for abrupt changes in climatic factors between northern and southern China. (a: Northern region; b: Southern region).

The M-K test revealed long-term monotonic trends and their significance in climatic factors across northern and southern China. However, it proved ineffective in pinpointing critical turning points in climate fluctuations. Therefore, Mutational analysis was conducted on various temperature-representative factors for northern and southern China between 1912 and 1949, with results presented in Fig 2. It is evident that the climates of northern and southern China underwent distinctly divergent breakpoint trajectories: In the northern region (Fig 2a), five points satisfied the criterion of an intersection point lying between the critical line U₀.₀₅ = ±1.96, with the UF curve beyond the intersection exceeding this critical line. These were: an abrupt increase in annual mean temperature difference (X5) in 1912, 1918, and 1934; an abrupt rise in annual mean precipitation (X1) in 1913 and 1946. This indicates that abrupt changes in the northern region during this period were primarily characterised by temperature difference oscillations and precipitation fluctuations, reflecting a climate exhibiting unstable, phased variations. In the southern regions (Fig 2b), only two distinct abrupt change points are evident: the increase in annual mean low temperature (X4) in 1918 and the rise in annual mean high temperature (X3) in 1934. These abrupt changes were characterised by significant warming, representing a clear warming transition within the climate system. This disparity profoundly reflects the complexity of China’s climate system: the north is more influenced by variations in the westerlies and precipitation systems. At the same time, the south responds more sensitively and directly to global or large-scale warming signals.

#### 3.2.3. Theil-Sen Median.

**Fig 3:**
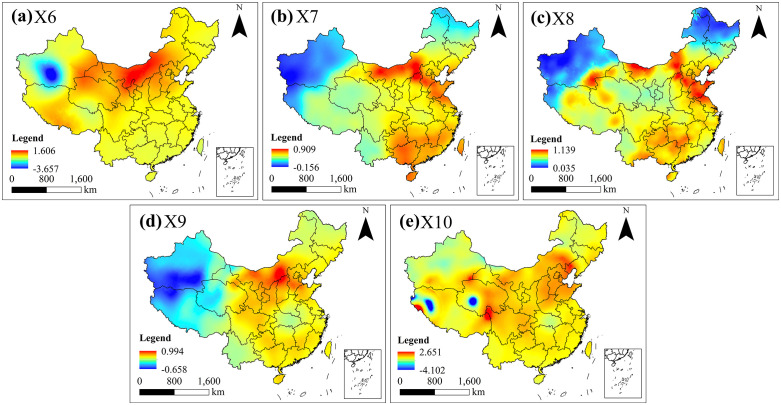
Trends in Chinese climate factors during the ROC period. The data used in this study is from the National Earth System Science Data Center (http://www.geodata.cn). The base map data for this study is sourced from the standard map, available at https://www.webmap.cn/mapDataAction.do?method=forw&datasfdafd=%253Fmethod%253Dforw%2526resType%253D5%2526dName%253D%2525E6%2525AF%252594%2525E4%2525BE%25258B%2525E5%2525B0%2525BA%2526format%253Dcombox%2526name%253DidMapScale%2526value%253D1000000%2526secClass%253D%2525E5%252585%2525AC%2525E5%2525BC%252580. The base map has not been modified in any way.

By presenting climate change trends across the entire territory of China through interpolation, it was found that these trends exhibit distinct regional differentiation (Fig 3). Fig 3a illustrates the trend in annual mean precipitation for 38a, revealing a significant increase in precipitation in the Inner Mongolia region of northern China, alongside a marked decrease in the Taklamakan Desert region of Xinjiang. The spatial patterns of annual mean temperature, annual mean maximum temperature, and annual mean minimum temperature trends were broadly consistent. Areas exhibiting warming trends were primarily concentrated in an arc-shaped high-value belt stretching from eastern Inner Mongolia to Shandong (Fig 3b, 3c, and 3d). This belt separated plague hotspots in northern regions, suggesting these three temperature factors may inhibit plague spread by forming an ecological isolation barrier. Additionally, high-value zones encompass southern regions including Guizhou, Jiangxi, Guangxi, Guangdong, Hunan, and Fujian. In contrast, low-value zones are observed across most of Xinjiang, Tibet, Heilongjiang and Jilin. The spatial distribution of high-value zones for annual mean temperature difference largely mirrors other temperature variables, appearing in eastern Inner Mongolia to Shandong and parts of southern China. However, low-value zones are situated in Qumalai County in Qinghai and Geji County in Tibet (Fig 3e).

### 3.3. Analysis of climate change impacts on plague outbreaks

**Fig 4:**
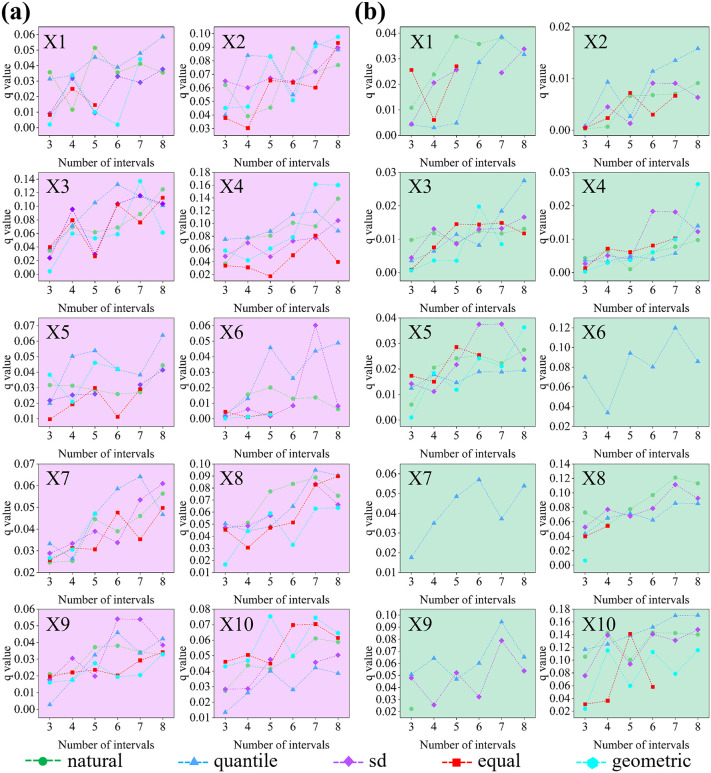
Optimised parameters for different factors in northern and southern China.

Optimisation of discrete parameters for climate factors revealed significant variations in optimal parameter combinations and breakpoint numbers across different factors (Fig 4). Fig 4a and 4b present the optimised parameter results for climate factors in northern and southern regions. The optimal parameter for each factor is the maximum value among the five discretization methods at different classification levels. Taking the annual average temperature in northern regions (X2) as an example, the maximum q-value occurs when the classification level is 8. At this point, the q-values of the five discretization methods are ranked as follows: Geometric Interval Method (0.098)> Equal Interval Method (0.093)> Standard Deviation Method (0.089)> Quantile Classification Method (0.088)> Natural Breakpoint Method (0.077). This indicates that when the number of categories is 8, the explanatory power of the geometric breakpoint method is significantly higher than that of other classification methods. The specific values of the optimal parameters for other factors can be found in the S1 Appendix. After integrating all factor data for unified analysis, the optimal parameter combination for X1 and X5 in the Northern region selected the quantile classification method based on 8 breakpoints. The optimal parameter combination for X7 and X8 was the quantile classification method based on 7 breakpoints. The optimal parameter combination for X3 and X4 was the geometric interval method based on 7 breakpoints. The optimal parameter combinations for X2 and X10 were the geometric interval method based on 8 and 5 breakpoints, while those for X6 and X9 were the standard deviation method based on 7 and 6 breakpoints. For the southern region, the optimal parameter combinations for X1 and X8 are the natural breakpoint method based on 5 and 7 breakpoints; for X2 and X3, it is the quantile classification method based on 8 breakpoints; for X6, X9, and X10, it is the quantile classification method based on 7 breakpoints; The optimal parameter combination for X7 is the quantile classification method based on 6 breakpoints. The optimal parameter combination for X4 is the geometric interval method based on 8 breakpoints. The optimal parameter combination for X5 is the standard deviation method based on 7 breakpoints.

**Fig 5:**
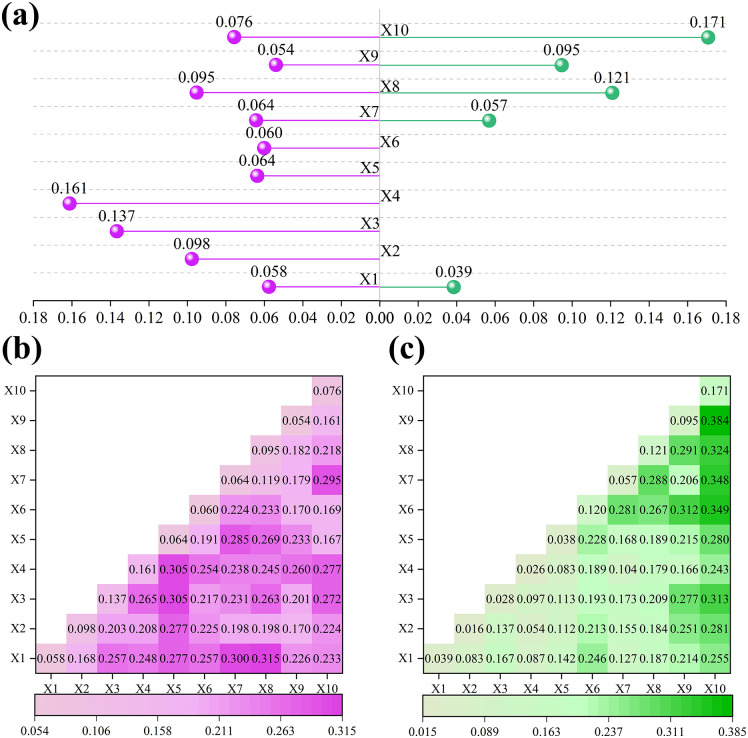
Results of interactive detection of plague influencing factors in northern and southern China.

An OPGD investigated factors influencing plague incidence in northern and southern China. Fig 5 presents the computational results of the optimal parameter geographic detector. Fig 5a displays q-statistic results for the single-factor effects of plague influencing factors across northern and southern China. When the p-value of the measurement result is less than 0.05, it indicates that the factor is statistically significant and can provide a certain degree of explanatory power for plague occurrence. Comparative analysis reveals that in the northern region, all ten climatic factors exhibit associations with plague incidence, with X4 (annual mean low temperature) demonstrating the strongest explanatory power at 16.12%. Conversely, only five climatic factors in the southern region show correlations with plague, where X10 (annual temperature variation trend) exhibits the highest explanatory power at 17.07%.

Fig 5b and 5c present the interaction results for plague influencing factors in northern and southern regions. Interactions between plague-influencing factors generally exceed the effects of individual factors, indicating that climatic factors in northern and southern China do not act independently on plague incidence but exhibit specific nonlinear interactions. In northern regions, interaction detection results indicate that the combination of annual precipitation versus annual high temperature trends and annual precipitation versus annual temperature trends exhibits stronger spatial stratification heterogeneity explanatory power, with interaction Q-values of 31.46% and 30.01%, respectively. In northern regions, interaction detection results indicate that the combination of annual mean precipitation ∩ annual mean high temperature trends and annual mean precipitation ∩ annual mean temperature trends exhibits stronger spatial stratification heterogeneity explanatory power, with interaction q-values of 31.46% and 30.01%, respectively. In southern regions, interaction detection results indicate that the combinations of annual mean temperature difference trend ∩ annual mean low temperature trend, annual mean temperature difference trend ∩ annual mean precipitation trend, and annual mean temperature difference trend ∩ annual mean temperature trend exhibit stronger explanatory power for spatial hierarchical heterogeneity, with interaction q-values of 38.44%, 34.92%, and 34.77%, respectively.

### 3.4. Spatiotemporal coupling between climate change and plague

**Fig 6:**
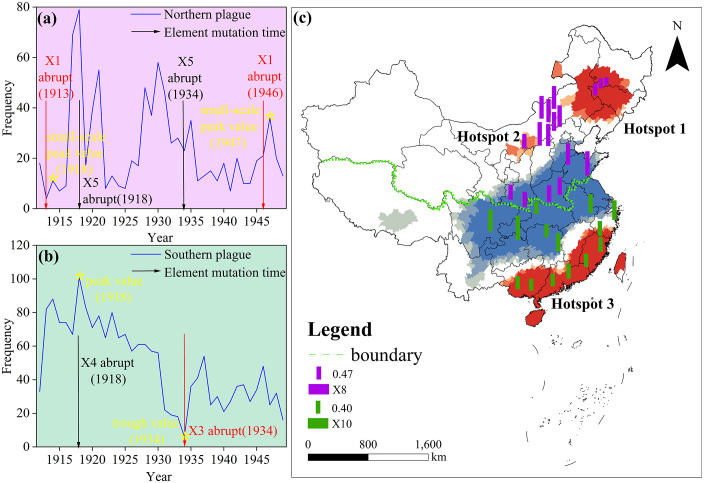
Spatiotemporal coupling between climate change and plague in northern and southern China. The data used in this study is from the National Earth System Science Data Center (http://www.geodata.cn). The base map data for this study is sourced from the standard map, available at https://www.webmap.cn/mapDataAction.do?method=forw&datasfdafd=%253Fmethod%253Dforw%2526resType%253D5%2526dName%253D%2525E6%2525AF%252594%2525E4%2525BE%25258B%2525E5%2525B0%2525BA%2526format%253Dcombox%2526name%253DidMapScale%2526value%253D1000000%2526secClass%253D%2525E5%252585%2525AC%2525E5%2525BC%252580. The base map has not been modified in any way.

Analysis of the spatiotemporal coupling between climate change and plague outbreaks (Fig 6) reveals distinct temporal response patterns between northern and southern regions to abrupt climate shifts. In the northern regions (Fig 6a), abrupt changes in precipitation (X1) (such as those occurring in 1913 and 1946) typically triggered minor peaks in plague incidence within the subsequent 1–2 years (1914 and 1947). Conversely, abrupt shifts in annual mean temperature difference (X5) were generally associated with declining plague incidence. In southern regions (Fig 6b), years of abrupt changes in annual mean high temperatures (X3) corresponded to the lowest plague incidence, followed by a rapid rebound. Conversely, years of abrupt changes in annual mean low temperature (X4) aligned with peak plague incidence, which declined sharply within one year. Spatially, the spatial coupling results between plague frequency and climate factor trends reveal (Fig 6c) that low-value zones of the annual mean high temperature trend in northern regions highly overlap with plague hotspots. In contrast, its high-value zones form ecological barriers isolating adjacent hotspots. Southern plague hotspots predominantly cluster within low-value zones of the annual mean temperature difference trend, whereas high-value zones predominantly exhibit plague cold spots.

## 4. Discussion

This study demonstrates a significant spatiotemporal coupling effect between climate change and plague epidemics, with fundamentally distinct response mechanisms in northern and southern regions. The key findings are discussed in detail below.

### 4.1. Spatiotemporal divergence in plague epidemics between north and south stems from differing climate drivers and ecological contexts

The elaboration of climate interactions by OPGD is used here to statistically verify the superposition mechanism of many nonlinear effects between plague drivers simulating across regions in China and to uncover deeper bioecological drivers. In the northern zone, strong interactions between precipitation and the high-temperature trend (q = 31.46%) reflect a typical substrate control mechanism in semiarid zones. In other words, as a key limiting factor, fluctuation in precipitation alleviates food limitations and promotes vegetation growth to increase host densities; temperature then promptly speeds up the life history of vector fleas hitting high host density based on high transmission risk [25]. to be translated into a cascade effect. Indeed, this is the reason for the poor explanatory power of individual factors and that their joint effects contribute to more than 30% of the globe’s spatial variation. In the southern area, the driving mechanism primarily has to continuously provide environmental carrying capacity. Strong interaction of temperature difference and cold trend (q = 38.44%) indicates that plague incidence is sensitive to environmental variation. The increase in the low-temperature trend (warm winters) weakens low-temperature barrenness on flea overwintering success. At the same time, this trend of stability in the temperature difference contributes to uninterrupted annual circulation [10]. In other words, the main climate driver is warmth that dissipates ecological bottlenecks by stretching the epidemic focus into more extensive spatial and temporal dimensions.

### 4.2. Climate abruptions act as ‘switches’, triggering phased transformations in plague epidemic patterns

Time-coupling analysis reveals the critical ‘trigger’ role of climate abruptions. In the north, minor plague peaks emerge 1–2 years after precipitation abruptions, consistent with the ‘precipitation-driven’ conclusion. The likely mechanism involves precipitation abruptions improving the survival environment for vector fleas or host animals, leading to enhanced plague activity after one reproductive cycle [26]. In the south, years of abrupt annual high temperature shifts corresponded to plague lows, followed by increased frequency. This seemingly contradictory phenomenon may signal a climate system reorganisation: based on research by Cavanaugh [27] and others, environmental temperatures above 27.5°C suppress expression of the plague bacterium’s hms locus, blocking its ability to form biofilm plugs in the flea’s forestomach and thereby interrupting the transmission chain. Simultaneously, extreme heat stress directly increases flea larval mortality through dehydration [28]. Thus, sporadic warming events reduce plague activity in the short term by diminishing both pathogen virulence and vector population density. However, as the climate reaches equilibrium in a new state (completing a ‘warming jump’), the plague system adapts and reorganises, establishing new epidemiological patterns [10]. This finding indicates that predicting plague epidemics requires attention to the steady-state values of climatic factors and the critical threshold behaviour of their abrupt transitions.

### 4.3. Spatially coupled results reveal how climate trends shape the geographical boundaries of plague distribution

The spatial distribution of climate trend changes, coupled with the association with plague hotspots and cold spots, has succeeded in its new rationale for expanding or even focal stability in focus dynamics. In this respect, the north conceptualization of the ecological barrier built up of ‘annual mean temperature high-value areas’ is directly linked to the physiological mechanism underlying heat stress on cold-resistance hosts: that is, the core northern focal hosts, such as marmots, drastically reduced the thermal window for foraging during the day under rapid warming of climate. Under warm temperature conditions, marmots would reduce their exposure above ground to avoid the heat, thus working against accumulating pre-hibernation fat, thereby significantly increasing winter mortality and resulting in a “geographical fault” in the population density [11]. At the same time, high temperature during the development of the flea was generally accompanied by low relative humidity, thus greatly enhancing larval flea rate mortality by desiccation and reducing adult flea longevity. Above 28°C, the thrombolysis of bacterial plugs formed in the flea’s fore-stomach rapidly reduces the effective transmission potential of these vectors. Thus, these two factors thermal compression of the host domicile and dynamic disruption of the transmission chain work jointly to make a natural transmission barrier [29]. In other words, this implies that rapid climate warming has contracted the habitat of the plague reservoir host marmots, thus fragmenting the original foci [30]. Conversely, in the south, ‘low-value zones of trend in annual mean temperature correspond to hotspots,’ indicating that regions with stabilising diurnal temperature fluctuations may better sustain intact plague transmission chains [31]. This finding suggests climate trends may geographically ‘carve’ plague distribution patterns by altering habitat suitability.

### 4.4. Dialogue with prior research and future prospects

In comparison with other studies (e.g., Xu et al.) [10,11], which focused on the nonlinear response curves of plague epidemics to temperature and precipitation in China, the current study focused on identifying two fundamentally different driving mechanisms in northern and southern China through the OPGD method. A short-term pulse-driven mechanism was identified in the north, while a long-term trend-driven mechanism was detected in the south. This further explains why plague epidemics can be better explained by the interaction of climate factors than by a single factor. The cooperative driving logic is better than the logic of single factors such as temperature or precipitation proposed by Xu, which can better reflect the complexity of plague epidemics in nature. Meanwhile, in our study, it becomes evident that phases of plague change (delayed peaks after precipitation change in the north, for example) were initiated after abrupt changes in climate. On a spatial dimension, areas with high annual temperature trends become ecological barriers to isolate plague hotspots in the north. A spatiotemporal association analysis adds a spatial dimension to the argument based on the nonlinear functions that Xu proposed.

Hence, the results from this study provide important supplementary information to such short-time studies or case studies focused at specific plague foci. This study reveals a highly regionally dependent climate-driven plague dynamic, which has also been observed for other large international foci. For example, the desert foci in Central Asia are strongly driven by the trophic cascade effect through climatic influences on precipitation and temperature [25], while the US plague risk is linked to strong large-scale responses in precipitation anomalies induced by the El Niño-Southern Oscillation, affecting flea overwintering and host breeding conditions through changes in near-surface microclimate moisture [32]. Compared with the above-described studies, this research interestingly divided the dynamics of the plague-burdening double plague-bringing patterns into the two regions, the northern portion versus the southern portion, and the “mean/short-term fluctuation-driven” versus the “long-term trend accumulation-driven,” thus a new dimension to understanding the influence of climate on disease evolution, further strengthening the view that plague epidemics are more likely to result from joint and nonlinear effects of multiple climate factors-that is, more plausible scenarios of plague epidemic manifestations.

### 4.5. Prevention recommendations

Establish a dynamic plague zoning early warning system: break the nationwide same mold; to the North, attach importance to the lag hazard caused by extreme precipitation, and attach importance to the lag hazard-induced by sudden heavy rainfall in arid areas within 1–2 years; to the South, attach importance to the outward expansion hazard of plague foci under the long-term stable rising trend of temperature. Identify the high-risk interaction time; the dynamic analysis should not be limited to the situation where the number of single factors is too high, but the dynamic analysis should be associated with the interaction time of factors, such as “continued high precipitation + sudden high temperature,” which is one of the conditions for upgrading alarms. Relying on the function of climate barriers in the north, ecological barriers caused by temperature increases may refer to the layout of the density of plague monitoring stations: in plague foci affected by climate change, dynamic tracking and monitoring of *Yersinia pestis* reservoir habitat shifts may need to be further strengthened.

Limitations in applying this research: First, plaquesive data and historical plague-data have been evaluated in construction and accuracy, but multilayered grassroots administrative effectiveness and reporting systems throughout various historical epochs could still underlie the recording bias. That is not so much because the empirical study focuses on exploring relative spatio-temporal fluctuation patterns and differentiation of driving mechanisms related to climate rather than absolute incidences; such a perspective means that systematic bias would significantly reduce the plague’s overall incidence relative to the logic of derivation concerning climate-disease dynamics. Second, epidemics are the result of both social and natural factors; for example, the transportation network would reduce space-time distance and thus change the spatial linkage between epidemic sources, speeding up the spread of plague. In this case, more comprehensive development in future research will include socioeconomic indicators such as population density, transportation networks, and epidemic prevention measures to build a “human-landscape-disease” coupled system model for simulation and prediction of potential plague risks.

## 5. Conclusions

(1) From 1912 to 1949, significant disparities existed in the incidence frequency of plague between northern and southern China. Southern plague was characterised by ‘wide fluctuations and stable trends’, exhibiting a clear downward trajectory. Northern plague, while showing an overall decline, was marked by ‘substantial fluctuations and modest reductions’, lacking a discernible decrease pattern. Spatially, the provincial distribution of plague incidence in the north ranked as follows: Inner Mongolia 28.82% > Jilin 18.48% > Shanxi 13.42% > Heilongjiang 7.59%. In the south, the provincial distribution ranked: Fujian 39.71% > Guangdong 23.54% > Guangxi 19.45% > Yunnan 5.37%.
(2) Significant differences exist between plague hotspots and coldspots: Hotspot 1 is located at the junction of Heilongjiang, Jilin, and Inner Mongolia, centred on Tongliao City, Da’an City, and Tongyu County; Hotspot 2 lies in the agro-pastoral transition zone of western Inner Mongolia to northern Shaanxi, primarily distributed around Ordos; Hotspot 3 is concentrated in the coastal areas of southeastern China, mainly southern Guangxi, Guangdong, and Fujian provinces. Concurrently, these hotspot regions highly overlap with plague foci.
(3) From 1912 to 1949, climate fluctuations in northern and southern China exhibited considerable variability. While southern precipitation declined, other climatic factors increased across both regions. Precipitation fluctuations and temperature difference oscillations characterised northern abrupt changes; southern abrupt changes centred on significant warming, representing a clear climatic warming transition. Significant regional disparities existed in the trends of annual mean high temperatures and annual temperature differences, with their spatial heterogeneity exerting a regulatory influence on the spatial pattern of plague epidemics.
(4) Analysis using the OPGD indicated that interactions between influencing factors generally exerted a more substantial impact on plague occurrence than the effects of individual factors alone. In northern regions, the core drivers are the ‘annual mean precipitation ∩ trend in annual high temperature’ (q value:31.46%) and the ‘annual mean precipitation ∩ trend in annual mean temperature’ (q value:30.01%), driven by the interaction between annual mean precipitation and trends in high temperature and mean temperature. In southern regions, the dominant factors were ‘trend in annual mean temperature difference ∩ trend in annual mean low temperature’ (q value:38.44%), ‘trend in annual mean temperature difference ∩ trend in annual mean precipitation’ (q value:34.92%), and ‘trend in annual mean temperature difference ∩ trend in annual mean temperature’ (q value:34.77%), reflecting synergistic interactions between annual mean temperature differences and other climatic variables.
(5) Temporal coupling results indicate that in northern regions, minor plague peaks emerged 1–2 years following abrupt annual precipitation shifts (1913 and 1946), while southern regions exhibited plague lows corresponding to abrupt annual high temperature shifts (1934). Spatial coupling results indicate that low-value zones of the annual mean high temperature trend in northern regions correspond to plague hotspots. In contrast, high-value zones form ecological barriers segmenting, adjacent hotspot areas. In southern regions, low-value zones of the annual mean temperature difference trend correspond to plague hotspots, whereas high-value zones correspond to plague cold spots.

## Supporting information

S1 Appendix Supplementary figure containing full details about the OPGD model and its parametrization, as well as additional results. (XLSX)

S1 Data Data of plague and climate factors in the north and south of China. (XLSX)
